# Quantitative ELISAs for serum soluble LHCGR and hCG-LHCGR complex: potential diagnostics in first trimester pregnancy screening for stillbirth, Down’s syndrome, preterm delivery and preeclampsia

**DOI:** 10.1186/1477-7827-10-113

**Published:** 2012-12-17

**Authors:** Anne E Chambers, Christopher Griffin, Samantha A Naif, Ian Mills, Walter E Mills, Argyro Syngelaki, Kypros H Nicolaides, Subhasis Banerjee

**Affiliations:** 1 Department of Clinical Biochemistry, Heartlands Hospital, Birmingham, B9 5SS, UK; 2 MUMS Clinic, Solihull, Birmingham, UK; 3 Birmingham Women’s Hospital, Edgbaston, Birmingham, UK; 4 Harris Birthright Research Centre for Fetal Medicine, King’s College Hospital, London, UK; 5 Present address: Origin Biomarkers, BioPark, Broadwater Road, Welwyn Garden City, Hertfordshire, AL7 3AX, UK

**Keywords:** LHCGR, ELISA, Down’s syndrome, Stillbirth, Preeclampsia, Preterm delivery, Early pregnancy

## Abstract

**Background:**

Soluble LH/hCG receptor (sLHCGR) released from placental explants and transfected cells can be detected in sera from pregnant women. To determine whether sLHCGR has diagnostic potential, quantitative ELISAs were developed and tested to examine the correlation between pregnancy outcome and levels of serum sLHCGR and hCG-sLHCGR complex.

**Methods:**

Anti-LHCGR poly- and monoclonal antibodies recognizing defined LHCGR epitopes, commerical anti-hCGbeta antibody, together with recombinant LHCGR and yoked hCGbeta-LHCGR standard calibrators were used to develop two ELISAs. These assays were employed to quantify serum sLHCGR and hCG-sLHCGR at first trimester human pregnancy.

**Results:**

Two ELISAs were developed and validated. Unlike any known biomarker, sLHCGR and hCG-sLHCGR are unique because Down’s syndrome (DS), preeclampsia and preterm delivery are linked to both low (less than or equal to 5 pmol/mL), and high (equal to or greater than 170 pmol/mL) concentrations. At these cut-off values, serum hCG-sLHCGR together with PAPP-A detected additional DS pregnancies (21%) which were negative by free hCGbeta plus PAPP-A screening procedure. Therefore, sLHCGR/hCG-sLHCGR has an additive effect on the current primary biochemical screening of aneuploid pregnancies. More than 88% of pregnancies destined to end in fetal demise (stillbirth) exhibited very low serum hCG-sLHCGR(less than or equal to 5 pmol/mL) compared to controls (median 16.15 pmol/mL, n = 390). The frequency of high hCG-sLHCGR concentrations (equal to or greater than 170 pmol/mL) in pathological pregnancies was at least 3-6-fold higher than that of the control, suggesting possible modulation of the thyrotropic effect of hCG by sLHCGR.

**Conclusions:**

Serum sLHCGR/hCG-sLHCGR together with PAPP-A, have significant potential as first trimester screening markers for predicting pathological outcomes in pregnancy.

## Background

Human chorionic gonadotrophin (hCG) operates as the “Master Regulator” of human pregnancy: blastocyst development, implantation, vascular remodeling, placental invasion, maternal immunosupression at early pregnancy and fetal development are contingent upon various hCG functions [1]. The cellular signaling transduced by hCG, however, is dependent upon its cognate receptor LHCGR expressed in the placenta, fetus, gonads, reproductive tract and in a variety of non-gonadal tissues [2]. Unlike hCG, very little is known about how LHCGR modulates its ligand activities in human pregnancy. Moreover, it is also unknown whether LHCGR through cognate and non-cognate ligand interactions could regulate the thyrotropic effect of hCG [3] at early human pregnancy [4]. The conventional animal model (mouse), which is incapable of producing hCG, is ineffective in addressing LHCGR dynamics relevant to human pregnancy.

LHCGR is a G-protein coupled receptor with leutenizing hormone (LH)/hCG-binding sites at the N-terminus extracellular domain (ECD), six transmembrane (TM) domains and short intracellular C-tail [5]. In addition to mature LHCGR protein, multiple truncated natural variants are produced as a result of alternative splicing [6]. The cell-free soluble LHCGR (sLHCGR) has been detected in follicular fluid [7] and as hormone-receptor complex in the Leydig cell culture media [8]. Moreover, cells transfected with naturally truncated rat [9] and porcine [10,11] Lhcgr variants resulted in the secretion of Lhcgr and hCG-Lhcgr complex proteins into the culture media. Recent studies [12] revealed that in addition to the mature LHCGR (M_r_, 85-90K), the microvesicles released from the placental explants under stress contained two additional receptor variants (M_r_, 52K and 62K). LHCGR-antibody affinity purification of proteins from early human pregnancy serum resulted in the detection of three proteins with Mr 50K, 62K and 85K in western blots (unpublished observations).

The secretion of the soluble LH/hCG receptor from cultured transfected cells [8-11], placental explants [12] as well as the identification of a circulating LHCGR inhibitor protein in serum [13] raised the necessity of investigating the presence of sLHCGR or LH/hCG-sLHCGR complexes in the blood serum or other body fluids. However, the absence of a simple and inexpensive experimental system for a large-scale quantitative analysis of sLHCGR in human blood prevented such investigations. Here we describe the development of two ELISAs that specifically measure sLHCGR and the hCG-sLHCGR receptor complex in human serum and their applications in prenatal, first trimester screening for Down’s syndrome, fetal demise, preeclampsia and preterm birth.

## Methods

### Antibodies

Purified LHR29 and LHR74 antibodies were initially provided by Dr Hugues Loosfelt (INSERM, France) and subsequently the antibody producing clones were obtained from ATCC (Clone ID CRL-2685 and CRL-2686). Antibodies produced in mouse ascites fluid were purified by Protein A affinity chromatography. The PG732 is a goat polyclonal antibody raised against a 19 residue LHCGR peptide (LHCGR 209–227; Swissprot: locus LSHR_HUMAN, accession P22888). The same peptide was used for affinity purification of the antibody from ammonium sulphate precipitated PG732 immune goat serum and was also used to produce mouse monoclonal antibody clone 5A10C9. Therefore, 5A10C9 is a monoclonal version of the PG732 goat anti-LHCGR polyclonal antibody; LHR H-50 (rabbit) and LH K-15 (goat) antibodies against the N-terminus and internal region of human LHCGR respectively, were from Santa Cruz Biotech, USA; anti-hCGbeta monoclonal antibody (clone 094–10627) was from Acris, Germany; anti-FLAG peptide monoclonal antibody was from Sigma-Aldrich (USA). A variety of monoclonal antibodies against hCG or hCGbeta from various commercial sources were also tested during the course of assay development. Antibodies were conjugated using a Lightning Link horse-radish peroxidase (HRP) conjugation kit (Innova Biosciences, Cambridge, UK) and stored at 4°C for up to five months.

### Expression, cell extraction and affinity purification of recombinant LHCGR peptides in CHO cells, and western blotting of recombinant LHCGR and hCGbeta-LHCGR protein standards for ELISA

Details of the construction of LHCGR cDNA clones, transfection of Chinese hamster ovary (CHO) cells and expression of the recombinant proteins have been recently described [12]. Briefly, three LHCGR recombinant proteins with 3X FLAG epitope tagged at the C-termini and containing 229, 291 and 318 amino acids of the LHCGR ECD were independently expressed in CHO cells in suspension culture. Following 48h of transfection, the recombinant proteins were extracted either with lysis buffer (25 mM Hepes pH 7.5, 150 mM NaCl, 1% igepal CA-630 [Sigma-Aldrich], 10 mM MgCl_2_, 1 mM EDTA, 25 mM NaF, 1 mM Na_3_VO_4_, and EDTA-free protease inhibitor mix [Sigma-Aldrich] or M-PER reagent [Perbio, Helsinborg, Sweden]. For western blot analysis of the extracts, anti-FLAG, LHR29, LHR74 primary antibodies were diluted at a concentration of 1 μg/mL; PG732 and LHR-H50 were diluted 1 in 5000 and 1 in 2,000, respectively. Both Triton and M-PER lysed extracts expressing LHCGR291 were used for the development of the ELISA assays; however, Triton-lysed extracts were consistently better than the M-PER extracts. The recombinant protein (LHCGR291) was affinity purified using anti-FLAG M2 affinity column according to the protocol provided by the vendor (Sigma Aldrich, USA). The preparation of placental extracts from early pregnancy (12 wks) for western blots were as described [12].

### The sLHCGR and yoked hCG-sLHCGR complex protein standards for ELISA

In order to establish the specificity of the sLHCGR ELISA assay, the LHCGR291 recombinant and mock (control) transfected CHO cell extracts were initially employed. The LHCGR291 recombinant protein, which contains the hCG binding site and binds hCG in plate assays, was routinely used to generate standard curves with capture-detection antibodies as described below. However, the quantitative yield from transfected CHO suspension cell culture following anti-FLAG affinity purification was low (<600 μg/L). Therefore, following initial functionality tests in ELISA, LHCGR ECD was subsequently produced in bulk via a bacterial expression system. Other laboratories had shown that the expression of soluble LHCGR (sLHCGR, N-terminal 336 residues of the extracellular domain) as a thioredoxin fusion protein in *E*. *coli* carrying mutations in both thioredoxin reductase (*TrxB*) and glutathione reductase (*gor*) genes [14], had a similar specificity and affinity for hCG as the intact native LHCGR [15]. Therefore, this protocol was followed in order to produce the sLHCGR standard calibrator for ELISA assays. The protocol for expression of the sLHCGR fusion protein and affinity purification through Ni-NTA resin column (Qiagen) were exactly as described [15]. The estimated molecular mass of the fused sLHCGR was 57.54 K with pI of 6.15. The affinity purified protein was >90% pure and the yield varied from 6.63 to 7.34 mg/L.

For the production of the hCG-sLHCGR standard calibrator we employed a different method that aimed to preserve natural eukaryotic modifications of the hCG moiety of the final yoked protein. Previous studies [16] had shown that a tethered single chain hCG and LHCGR cloned in baculovirus and expressed in insect cells was functional with respect to ligand-receptor interaction. Moreover, the yoked hCG-LHCGR ECD is secreted from insect cells at levels 20-fold higher than conventional eukaryotic expression systems [16]. Our goal was to produce yoked hCG-LHCGR single chain protein containing the epitopes recognized by both hCGbeta and LHCGR antibodies. Therefore, the open reading frame encoding the entire 165 amino acids of hCGbeta was synthesized (ACCESSION AK291552; CGB165). A linker sequence encoding the hCGbeta C-terminal peptide (CTP, constituting amino acids 116–145 of hCGbeta) was ligated at the 3’ end of the above construct (CGB165-CTP). A cDNA clone encoding 115 to 291 amino acid residues of the N-terminal end of LHCGR ECD was produced. The CGB165-CTP was ligated to the 5’-end of the modified LHCGR cloned into p3XFLAG-CMV-14 vector to create 101235–1 clone. The hCGbeta-LHCGR complex was first expressed in transfected CHO cells. The specificity of the yoked hCGbeta-LHCGR protein was established by testing anti-LHCGR, anti-hCGbeta and anti-FLAG monoclonal antibody binding of the recombinant and mock transfected CHO extracts in plate assays as well as by western blotting. Following these initial functionality tests in ELISA, the yoked protein was subsequently produced in recombinant baculovirus transfected insect cells.

For baculovirus expression, the cDNA encoding hCGbeta-CTP-LHCGR (clone 101235–1) with C-terminal 3XFLAG was transferred to a baculovirus vector, DH10Bac strain was used for the recombinant bacmid (rbacmid) generation. The positive rbacmid containing 3xFLAG tagged 101235–1 was confirmed by PCR and the final clone was characterized by DNA sequencing. The rbacmid was transfected into an sf9 insect cell line, using Cellfectin, incubated in SF-900 liquid medium for 5–7 days at 27°C. The supernatant was collected and designated as P1 viral stock. P2 was amplified for later infection. The results of expression evaluation by western blot indicated that the target protein was expressed at the expected relative molecular mass. The recombinant protein was purified from the supernatant by loading on Flag M2 affinity gel, and the beads were eluted with TBS (50 mM Tris–HCl, 150 mM NaCl, pH7.4) containing 200 ng/μl peptide (FLAG: N-Asp-Tyr-Lys-Asp-Asp-Asp-Asp-Lys-C relative molecular mass, 1013.0). The expression and purification results indicated that the target protein was largely released from the cells into the supernatant. The yield of the yoked hCGbeta-LHCGR protein was 0.42 mg/L with an estimated purity of 70%. The affinity purified protein was stored in 20% glycerol at −20°C.

### ELISA assays

Ninety-six-well plates (C-well binding capacity 500ng/well, 8-strip, polystyrene; Greiner Bio-one, Germany) were coated with 100 μl per well of LHCGR transfected CHO extracts (for initial ELISA functionality tests) or with 3 μg/mL affinity purified LHR29 (sLHCGR assay) or 5A10C9 (hCG-sLHCGRassay) diluted in 50 mM Carbonate/Bicarbonate buffer pH 9.4 (Thermoscientific/Pierce, UK) at room temperature (RT) overnight. Following removal of the antibody, plates were over-coated with 300 μl per well of 10mM sodium/potassium phosphate buffer at pH 7.6 containing 5% sucrose (Fluka, UK) and 0.5% bovine serum albumin (Sigma-Aldrich, UK) for two hours at RT, before removal and drying overnight at RT. The pre-coated ELISA plates were wrapped and stored at RT and were stable for six months of use. On the day of use, plates were blocked for one hour at RT with 100 μl per well of phosphate buffered saline pH 7.2 (Thermoscientific/Pierce) containing 1% (v/v) casein concentrate (sdt reagents, Germany) prior to adding serum, antigen or standards diluted in 25 mM Bicine (Fluka), 50 mM Tris pH 7.8, 170 mM NaCl and incubation at RT for 2 h. Following binding, plates were washed six times with 300 μl per well 2 mM Tris Cl pH 7.8, 150 mM NaCl, 0.05% Tween 20 prior to incubation with HRP-conjugated antibodies (LHR29, 5A10C9 or anti-hCGbeta) diluted in Immunoshot 2 reagent (Cosmo Bio Co. Ltd, Japan) for 1h. The antigen-binding was detected by adding TMB (3,3^′^,5,5^′^-tetramethylbenzidine) substrate (Thermoscientific/Pierce, UK) and the color reaction was stopped by adding an equal volume of 1N HCl. Plates were read at 450-620 nm in a standard plate reader. Data were transferred to Microsoft Excel prior to analysis as described below.

### Patient serum samples

The major aim of this study was a prospective examination of the association of serum sLHCGR and hCG-sLHCGR concentrations at early human pregnancy (first trimester) with adverse pregnancy outcome. The study was approved by REC West Midlands, as part of National Research Ethics Services of NHS. Patient information and a patient consent form were given to each patient. As a standard Down’s syndrome screening requirement, pregnancies at 10–14 wks of gestation underwent ultrasound examination as well as nuchal scanning where indicated. A combined trisomy screen comprising biochemical analysis of free hCGbeta and PAPP-A was performed on all serum samples. As part of this standard screening, an aliquot of each serum sample from consenting patients, was stored at −20°C for further analysis of sLHCGR and hCG-LHCGR concentrations. A portion of the study was retrospective with regards to Down’s syndrome; 30 known T21 and 130 control samples (collected from 2006 to 2009) were obtained from the Fetal Medicine Foundation (FMF), UK. Unlike PS, the samples for the retrospective study (RS) were collected in a referral hospital designated for screening high risk fetal aneuploidy. Therefore, the samples for retrospective study obtained from FMF belonged to a high risk pregnancy group.

### Data analysis

The Analysis ToolPak (ATP) software was used to compute means, standard deviation (SD), variance (anova) and coefficient of variation (CV) for all data sets. For each ELISA assay, standard curve was generated following examination of the natural log plot of the optical density (OD) at 450-620 nm for all the standard points, following removal of the background diluent signal, to ensure that the points form a straight line. Using the scatter plot function, a standard curve was created using picomoles per mL of the standard as Y-value and OD (450-620 nm) on X-axis. By adding a line of best fit through zero, the equation and regression of a straight line (y = mx), representing the exponential, logarithmic portion of a standard curve, was generated. Generally, the regression, R^2^ >0.98, was considered valid. This standard curve and the dilution factors were used to measure the analyte concentration in a sample. Correlation testing was performed using the Pearson product moment method (standard R package). General file manipulations and data cleaning were implemented using the Awk programming language or custom programs written in Python. The graphical display of the distributions of data from control and pathological pregnancies were carried out using the ggplot2 package (R statistical software environment). The detection rates for pathological pregnancies were calculated as the proportion of pathological data points found to lie within the critical regions defined by the cut-off values set for two analytes. The false positive rate was calculated as the proportion of all control data points which were found within the critical regions defined by the cut-off values. The statistical significance of the difference in median values for hCGbeta and hCG-sLHCGR between control and pathological pregnancies were calculated using a Wilcoxon signed rank test in the R statistical software package since the data showed a strongly non-gaussian distribution.

## Results

### Epitope mapping of LHR 29 monoclonal antibody

Both LHR29 and LHR74 mouse monoclonal antibodies was raised against bacterially expressed extracelluar domain (ECD) and a part of TM domain of LHCGR containing amino acids residues 75–406 [17]. The specificity of LHR29 antibody was established by immunoprecipitation and immunopurification of ^125^IhCG-LHCGR complex, western blot analysis of the immunogen [17] and LHCGR ECD (amino acids 1–362) expressed in HEK 293 cells [18] and Dr Axel Themmen, personal communication.

**Figure 1:**
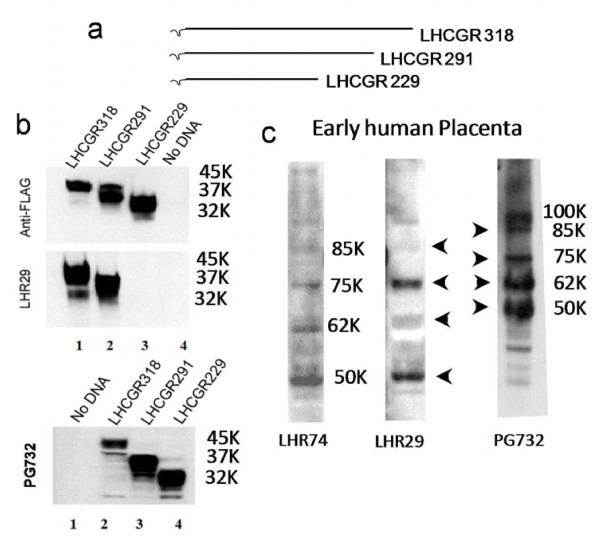
**Epitope mapping of LHR29 monoclonal antibody.a**) Three recombinant proteins containing 229, 291 or 318 amino acid residues of the LHCGR extracellular domain and C-terminal FLAG-epitope were expressed in CHO cells as described [12]; **b**), recombinant as well as mock transfected extracts (no DNA) were resolved in SDS-PAGE, blotted and probed with anti-FLAG, LHR29 and PG732 mono- and polyclonal antibodies respectively; **c**), two monoclonal antibodies (LHR74 and LHR29) and one polyclonal (PG732) were used in western blot analysis of proteins extracted from placental extracts at 12 wks of gestation.

To map the epitope for LHR29 monoclonal antibody (Mab), three recombinant LHCGR 318, 291 and 229 proteins fused with 3X-FLAG at the C-termini were expressed in CHO-S cells [12] and the extracts were probed with anti-FLAG and LHR29 monoclonal antibodies in western blots (Figure 1a). Unlike anti-FLAG Mab, LHR29 Mab failed to recognize LHCGR 229 protein while it reacted with LHCGR 318 and LHCGR 291 (middle panel). As an alternative positive control, a polyclonal antibody (PG732) raised in goat and affinity purified using a 19 amino acid residue LHCGR peptide (LHCGR residues 209–227) reacted with all three recombinants. Moreover, LHR H-50 (which recognizes an epitope corresponding to amino acids 28–77 within ECD of LHCGR, Santa Cruz Biotechnology, USA and ref [19]) recognized all three recombinants (data not shown). Together, these results showed that the epitope for LHR29 Mab resides within residues 229 and 291 amino acids of LHCGR ECD. 

Once the specificity of the LHR29 Mab and PG732 polyclonal antibody were established, the expression of the LHCGR protein was examined by western blot of placental extracts from early (12 wks of gestation) human pregnancy. The LHCGR monoclonal (LHR74 and LHR29) and polyclonal (PG732) antibodies were used as probes. In addition to mature LHCGR and high relative molecular mass (M_r_) proteins (85K and 100K), all antibodies reacted with M_r_ 50K, 62K and 75K isoforms (Figure 1b). While the same set of placental proteins were also recognized by LHR-H50 polyclonal antibody (Santa Cruz Biotech. USA), only mature (M_r_ 85-90K) and M_r_ 50K bands reacted with ^125^I-hCG (19). None of these bands reacted when the primary antibody was replaced by isotype-specific mouse IgG [18] and data not shown. Therefore, at the initial phase of this study LHR29 and PG732 were used as capture and detection antibodies, respectively, in ELISA for LHCGR measurement. Subsequently, the PG732 polyclonal antibody was replaced by 5A10C9 Mab.

### The specificity of the LHCGR standard for ELISA

**Figure 2:**
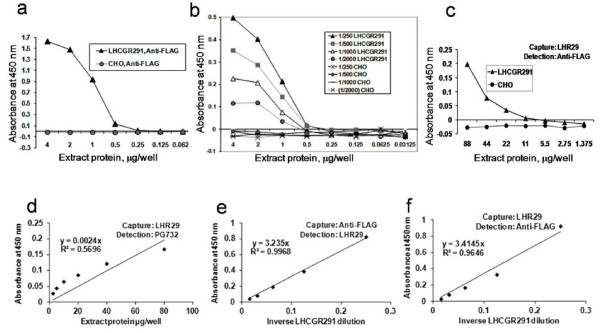
**The specificity of sLHCGR ELISA assay.** (**a**-**c**), demonstrates that LHCGR291 recombinant with C-terminal FLAG-tag specifically reacts with anti-FLAG and anti-LHCGR (LHR29) monoclonal antibodies; **a**) when plates coated with increasing amounts of LHR291 and CHO cell extracts were detected with anti-FLAG antibody and **b**) when plates coated with increasing amounts of LHR291 and CHO cell extracts were detected with serially diluted LHR29 antibodies or **c**), when plates coated with LHR29 antibody captured LHR291 in serially diluted extracts and was detected by anti-FLAG antibody: (**d**-**f**), the concentration-dependent reactivity of the LHCGR protein is shown where **d**) LHR29-PG732-HRP, **e**) anti-FLAG-LHR29-HRP and **f**) LHR29-anti-FLAG-HRP combinations were used as capture-detection antibodies respectively in ELISA assays specifically detecting LHCGR291 recombinant protein.

The LHCGR291 recombinant protein with C-terminal 3X FLAG tag (Figure 1a) which binds hCGbeta (data not shown) was tested either in cell extracts or as anti-FLAG affinity purified protein. The antigenic specificity of the recombinant was first verified by coating the plates with serially diluted LHCGR291 M-PER extracts that had been buffer exchanged using a PD10 column prior to coating, and reacting with anti-FLAG-HRP (Figure 2a) and serially diluted LHR29-HRP (Figure 2b). Moreover, the capture of LHCGR291 protein by LHR29 antibody, subsequently detected by anti-FLAG-HRP was sensitive to the extract protein concentration (Figure 2c). The lysates from CHO-S cells transfected with no DNA were incorporated as negative controls in these studies (Figure 2a-c). The specificity and sensitivity of *in vitro* affinity purified (anti-FLAG protein A sepharose) LHCGR291 recombinant as an ELISA standard were further tested. In these experiments, serially diluted LHCGR291 recombinant was captured and detected in three combinations (Figure 2d-e). The data shown in Figure 2d (LHR29-PG732-HRP), Figure 2e (anti-FLAG-LHR29-HRP) and Figure 2f (anti-FLAG-LHR29-HRP) revealed that linear standard curves could be generated in each condition. The specificity of the assay was established by a variety of controls including isotype-specific IgGs from rabbit, mouse and goat. These data led us to conclude that it was experimentally possible to produce recombinant LHCGR calibrator for quantitative measurement of sLHCGR in human serum by ELISA.

### The sLHCGR/hCG-sLHCGR protein standards, calibration and linear response to sample dilution effect

**Figure 3:**
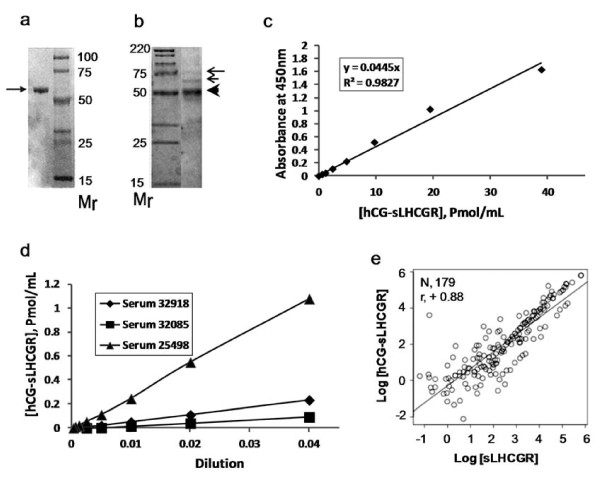
**The sensitivity and relationship of the sLHCGR and hCGbeta-sLHCGR ELISA assays.** The recombinant LHCGR and yoked hCG -LHCGR proteins together with anti-LHCGR and anti-hCG mono- and polyclonal antibodies provide quantitative standard curves in ELISA; **a**) and **b**), the coomassie-stained affinity purified recombinant human a) LHCGR ECD and b) hCGbeta-LHCGR proteins resolved in SDS-PAGE; c and d, both serially diluted protein standard **c**) and three serum samples from first trimester pregnancy **d**) exhibited linear response in ELISA assays where hCG-LHCGR complex was captured by 5A10C9 and was detected by HRP-conjugated anti-hCGbeta monoclonal antibody (clone 094–10627, Acris, Germany): **e**), shows strong positive correlation (r = 0.88) between sLHCGR and hCG-LHCGR when the analytes were measured in the same set of serum samples (N, 179).

The yield of anti-FLAG affinity purified recombinant LHCGR291 protein used to generate standard curves with capture-detection antibodies described above was 600–800 μg/L. Moreover, our best affinity purified LHCGR291 standard from cell extracts was 50-60% pure. Therefore, we turned to bacterially expressed affinity purified LHCGR ECD (see Methods) which consistently had >90% purity (Figure 3a). This LHCGR standard produced a linear response when LHR29 and 5A10C9 were used as capture and detection antibodies, respectively as described (12). Unlike sLHCGR, the hCGbeta tethered to amino acid residues 115–291 of the LHCGR ECD was expressed in insect cells and the subsequent anti-FLAG affinity purified fusion protein was ~60-70% pure (Figure 3b). Serially diluted hCGbeta-sLHCGR protein showed linear response when captured by 5A10C9 and was detected by HRP-conjugated anti-hCGbeta monoclonal antibody (Figure 3c). This ELISA assay, when tested using three, serially diluted, early pregnancy serum samples with known hCG-sLHCGR concentrations, showed linear responses to the dilution effect of each serum sample (Figure 3d). In order to establish the relation between the two assay systems, both sLHCGR and hCG-sLHCGR were measured in the same set of serum samples. The correlation coefficient (r) of the two assays was 0.88 (Figure 3e), suggesting that primary clinical evaluation of a large cohort study could be carried out with either one of the two assays. We have primarily used hCG-sLHCGR assays for clinical studies, because it provides a direct estimate of the amount of hCG bound to the circulating receptor.

### Sensitivity, precision and accuracy of the ELISA assays

In order to measure the sensitivity of the ELISA assays, the mean optical density of 16 duplicates of ‘zero standard’ (diluent) plus two standard deviations (SD) values in each case was extrapolated to the corresponding standard curve. The sensitivities of the sLHCGR and hCG-sLHCGR assays were 0.91 and 1.12 pmol/mL, respectively.

**Figure 4:**
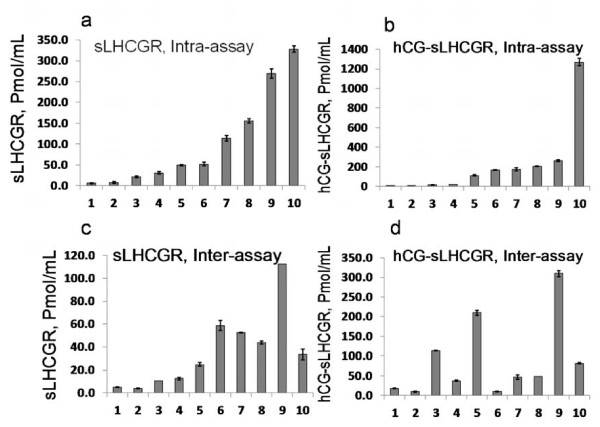
**Precision of the sLHCGR and hCG-sLHCGR ELISA assays.** Ten early pregnancy serum samples were used for intra- and inter-assay validation. Intra-assay variation of 10 sera, mean of octuplicates showing SD for sLHCGR (**a**) and hCG-sLHCGR (**b**). Inter-assay variation of 10 sera tested in duplicate one week apart showing SD for sLHCGR (**c**) and hCG-sLHCGR (**d**).

The intra-assay percentage coefficient of variation (CV%) was estimated by repeated measurement (n = 8) of 10 early pregnancy sera with known concentrations of sLHCGR (between 6 and 328 pmol/mL) and hCG-sLHCGR (between 3.4 and 1270 pmol/mL). The standard deviations of each sample for both analytes are shown (Figure 4a and b). The estimated CV% for serum samples with concentrations >10 pmol/mL were 2.3-10.4% and 2.8-9.3% for sLHCGR and hCG-sLHCGR, respectively.

The inter-assay CV% was estimated by repeated measurement (n = 4) of ten early pregnancy sera in two experiments carried out one week apart with identical sets of reagents and the same sera. The known sLHCGR and hCG-sLHCGR concentration range for the sera were 4.2-120 pmol/mL and 22–339 pmol/mL, respectively. The inter-assay CV% for sLHCGR and hCG-sLHCGR were 1.0-8.5% and 0.7-9.9%, respectively (Figure 4c and d).

To estimate the analytical recovery in ELISA assays, known concentrations of purified recombinant hCG-sLHCGR (3.89-15.56 pmol/mL) and sLHCGR (3.98-15.95 pmol/mL) were added to serum samples with known hCG-sLHCGR and sLHCGR concentrations, respectively. Serum samples (spiked or non-spiked) were assayed in duplicate and the mean recovery and SD of the analytes for each serum sample are shown in Table 1. The recovery of spiked hCG-sLHCGR at three different concentrations from six serum samples were 82.3-115%, with SD ranging from ±2.7 - ±17.2. The observed mean hCG-sLHCGR recovery from six samples (serum samples 1–6, Table 1) was 99.7% (SD, ±10.1). Unlike hCG-sLHCGR, the analytical recovery of sLHCGR (serum samples 7–9, Table 1) was between 52–74.7%. A closer examination suggested that matrix interference with spike recovery was dependent upon the analyte concentration. Reduced analyte recovery was observed irrespective of whether sLHCGR recombinant proteins were produced in bacteria or CHO cells, suggesting that variable recovery was independent of sLHCGR glycosylation and other eukaryotic protein modifications. The reduced spike recovery was not due to inhibitors in the matrix because the matrix effect was also observed in serially diluted serum. Since the spike recovery was high at low concentrations (4–8 pmol/mL, Sample 7–9, Table 1), the poor recovery at higher sLHCGR concentration could be caused by altered receptor conformation [20] resulting in protein aggregation. To distinguish the matrix effect on endogenous (serum) and exogenously added sLHCGR, the detection antibody was replaced with anti-FLAG-HRP which recognizes spiked sLHCGR only. This study showed that the detection of spiked and not serum sLHCGR was affected (data not shown). 

**Table 1:** Analytical recovery of defined concentrations of hCG-sLHCGR and sLHCGR spike into serum samples at three different known analyte concentrations

**Serum sample**	**Expected value Pmol/mL**	**Observed value Pmol/mL**	**Recovery %**	**Mean**	**SD**
1	3.89	3.88	99.74	106.94	± 11.37
	7.78	9.34	120.05		
	15.56	15.72	101.03		
2	3.89	3.66	94.087	113.07	± 17.24
	7.78	9.94	127.76		
	15.56	18.26	117.35		
3	3.89	2.94	75.5787	90.53	± 12.99
	7.78	7.71	99.1		
	15.56	15.08	96.915		
4	3.89	3.2	82.262	82.34	± 2.7
	7.78	6.62	85.09		
	15.56	12.4	79.692		
5	3.89	3.86	99.229	90.66	± 7.5
	7.78	6.63	85.219		
	15.56	13.62	87.532		
6	3.89	4.92	126.48	115.1	± 9.85
	7.78	8.5	109.25		
	15.56	17.05	109.58		
7	3.98	2.55	64.07	52	± 11.9
	7.97	4.13	51.82		
	15.95	6.43	40.313		
8	3.98	2.9454	73.857	49.6	± 23.5
	7.97	3.8396	48.139		
	15.95	4.2867	26.876		
9	3.98	3.3188	83.386	74.7	± 39.4
	7.97	8.692	109.06		
	15.95	5.0572	31.707		

Serum samples 1–6, hCG-sLHCGR; serum samples 7–9, sLHCGR.

### Quantitative measurement of serum hCG-sLHCGR in combination with PAPP-A increases the detection rate in primary Down’s syndrome screening

**Figure 5:**
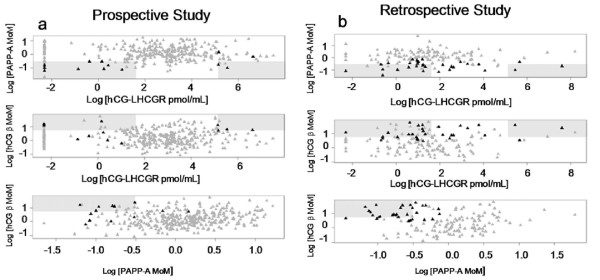
**Analysis of PAPP-A, free hCGbeta and hCGbeta-sLHCGR complex in trisomic and control pregnancy serum samples.** The distribution of PAPP-A, free hCGbeta MoM and hCG-LHCGR values (pmol/mL) from a prospective study (PS, n = 363) and a retrospective study (RS, n = 150) were plotted. The Down’s pregnancies (T21, n = 13 for PS and n = 30 for RS) in each plot are shown as dark closed triangles whereas the controls are shown by closed grey triangles in each plot. The cut-off values for PAPP-A and free hCGbeta detecting Down’s pregnancies were ≤0.5 MoM and ≥2.0 MoM respectively, as described [21]. The hCG-sLHCGR has low and high cut-off values. These values for detecting Down’s pregnancies were set at ≤5 pmol/mL and ≥170 pmol/mL, respectively. All data were log-transformed and the regions within these cut-off values in each plot are highlighted by shaded boxes.

For investigating the diagnostic potential of sLHCGR and hCG-sLHCGR in primary screening of trisomic pregnancies, the analyte concentrations in the early pregnancy sera (11–13.6 wks) from both prospective studies (PS) and retrospective studies (RS) were measured. The clinical data from the PS are shown in Table 2. Interestingly, chromosomally abnormal pregnancies could be detected at very low (≤5 pmol/mL) and at high (≥170 pmol/mL) concentrations, suggesting that hCG-sLHCGR is a unique biomarker and that within a certain range may have a physiological role in modulating normal pregnancy. The distributions of trisomy 21 (T21) and control pregnancies with combinations of two biochemical markers were plotted (Figure 5) and the detection rate (DR) and the corresponding false positive rates (FR) are shown in Table 3. A striking observation in these experiments was that >16% (7 of 43) Down’s syndrome pregnancies had extremely high circulating hCG-sLHCGR compared to those of euploid control pregnancies (5%, 24 of 470).

**Table 2:** Circulating hCG-sLHCGR in trisomic pregnancies

**Trisomy**	**hCGbetaMoM**	**PAPP-A MoM**	**NT MoM**	**T21 Risk**	**hCG-LHCGR Pmol/mL**
T21	0.99	0.24	3.50	2.00	0.32
T21	1.66	0.25	2.60	2.00	1.16
T21	1.94	1.08	2.60	24.0	173.73
T21	0.91	0.34	4.10	2.00	1.42
T21	2.05	0.75	3.40	7.00	759.59
T21	1.27	0.50	2.90	3.00	0.58
T21	3.04	0.35	1.90	2.00	0.00
T21	2.12	0.27	4.00	2.00	252.89
T21	2.92	0.36	1.40	63.0	170.74
T21	3.23	0.20	6.40	2.00	0.00
T21	3.92	0.50	1.0	38.0	1.07
*T21	2.829	0.281	3.1	2.00	0.00
T21	0.71	0.22	NA	2.00	2.67
*T17	7.65	2.22	NA	19.00	0.00
T18	1.16	0.14	3.00	2.00	4.36
T18	0.25	0.27	5.80	66.0	1.45
T18	0.78	0.37	NA	2.00	79.37

The multiplicity of median (MoM) for PAPP-A, hCGbeta, Nuchal translucency (NT), T21 risk are tabulated together with the hCG-sLHCGR concentrations. The ‘*’ and NA indicate live birth and data not available, respectively. T21, T17 and T18 are trisomy 21, 17 and 18, respectively.

**Table 3:** Detection rate (DR) and false positive (FP) rate in each analyte combination for prospective study (PS) and retrospective study (RS)

**Study type**	**No. Down’s**	**A**	**B**	**C**	**Additive effect on A’**
		**PAPP-A hCGbeta**	**PAPP-A sLHCGR**	**hCGbeta sLHCGR**	
**PS 350 + 13**	N = 13				
	DR%	46.1	84.6	53.84	38.46
	FP%	1.7	4.6	6	4.6
**RS 120 + 30**	N = 30				
	DR%	56.6	46.7	50	13.3
	FP%	0.83	4.16	13.3	4.16
**Total 470 + 43**	N = 43				
	DR%	53.5	58.13	51.1	20.9
	FP%	1.5	4.46	7.9	4.46

The cut-off values used for PAPP-A, hCGbeta and hCG-sLHCGR were ≤0.5 MoM, ≥2.0 MoM and ≤5.0 plus ≥170 pmol/mL, respectively. Additive Effect on A (PAPP-A and hCGbeta measurement) shows the percentage of additional T21 pregnancies detected by the hCG-sLHCGR and PAPP-A combination which had not been detected by PAPP-A and hCGbeta measurements.

In the prospective study, the detection rate of T21 using the biochemical combination PAPP-A plus hCGbeta alone was 46.1% with a false positive rate of 1.7%. In contrast, the detection rate of T21 using the biochemical combination PAPP-A plus hCG-sLHCGR alone, in the same prospective study, was 84% with a false positive rate of 4.6%. The detection rate of T21 (Down’s) in the retrospective study using the biochemical combination PAPP-A plus hCGbeta alone was 53.3% with a false positive rate of 0.83% However, using the biochemical combination PAPP-A plus hCG-sLHCGR for detection of T21 in the RS population did not increase the detection rate compared with the PAPP-A and hCGbeta markers. In the retrospective study population, the currently used combination of biochemical markers (PAPP-A plus hCGbeta) was more effective. These very different T21 detection rates for the PAPP-A and hCG-sLHCGR combination most likely reflect the differences between the two (PS and RS) populations. The prospective study contained individuals from the general population, whereas the retrospective study contained selected individuals at high risk of fetal aneuploidy. The detection rate by PAPP-A plus hCGbeta in prospective study (46.1% DR with 1.7% FP) reflects the expected results from a general population [21] rather than from the high risk samples selected from a retrospective study (56.6% DR with 0.83% FP). By combining the data sets from the prospective and retrospective studies, the PAPP-A plus hCGbeta detected 58.13% (23/43) of Trisomy 21 pregnancies with a false positive rate of 4.99%. However, a combination of PAPP-A and hCG-sLHCGR detected additional DS pregnancies (9/43) which were negative for the conventional PAPP-A plus hCGbeta combined screening. Therefore, quantitative measurement of hCG-sLHCGR together with PAPP-A and free hCGbeta increased the overall detection rate in the combined data sets from 58.13% to 72.1% (9/43 T21; 20.9% were additionally detected) with a false positive rate of 4.99%; suggesting that hCG-sLHCGR could be an effective marker for primary biochemical screening of Down’s syndrome in both low and high risk populations.

### Serum hCG-sLHCGR concentrations in preeclampsia, preterm delivery and stillbirth at early pregnancy

**Figure 6:**
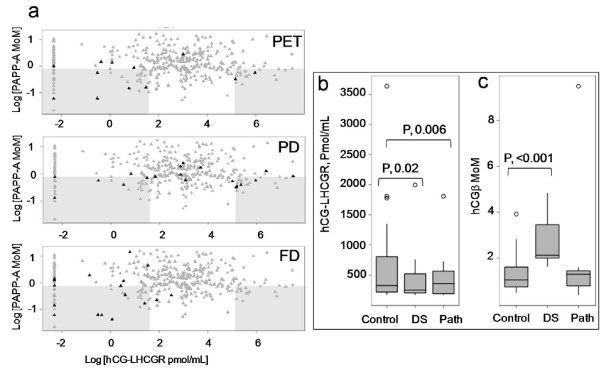
**Circulating hCG-sLHCGR in preeclampsia, preterm birth and fetal demise; a), the distribution of PAPP-A MoM and hCG-sLHCGR values (pmol/mL) from the prospective study (PS, n = 350) were plotted.** Adverse pregnancies (preeclampsia, PET; preterm delivery, PD and FD, fetal demise) in each plot are shown as dark closed triangles whereas the controls are shown by closed grey triangles. The cut-off value for PAPP-A was ≤0.8 MoM, while those for hCG-sLHCGRwere set at ≤5 pmol/mL and ≥170 pmol/mL. All data were log-transformed and the regions within these cut-off values in each plot are highlighted by shaded boxes; **b**) and **c**), the relationship between high serum hCG-sLHCGR(>170 Pmol/mL) and free hCGbeta with control (n = 30), Down’s syndrome (DS, n = 7) and other pathological (n = 11) pregnancies are shown.

Serum hCG-sLHCGR concentrations in pregnancies which were chromosomally normal but had pathological pregnancy outcomes were also investigated. The hCG-sLHCGR concentrations with respect to that of PAPP-A for preeclampsia (PET), preterm delivery (PD) and fetal demise (FD) are shown in Figure 6a. Unlike PET and PD, none of the pregnancies with fetal demise had high hCG-sLHCGR. However, both PET and PD had a pattern of hCG-sLHCGR profile very similar to that of Down’s syndrome. At ≤0.8 MoM and ≤0.5 MoM cut-off values for PAPP-A, the detection rates for PET were 58.3% and 41.6% with FP rate of 12% and 4.6%, respectively; at similar PAPP-A cut-off values, the detection rates for PD were 50% and 23.5% at the FD rates of 12.5% and 4.6%, respectively. At ≤0.8 MoM, the detection rate for FD was 52.3% with FP rate of 7.1%.

The prevalence of high levels of serum hCG-sLHCGR (≥170 pmol/mL) in controls, Down’s and pathological pregnancies, prompted examination of the relative concentrations of free hCGbeta in the same set of samples. The relative levels of hCG-sLHCGR and free hCGbeta in control, Down’s (DS) and pathological (Path) pregnancies are shown in Figure 6b and c, respectively. The median hCG-sLHCGR concentrations in control and pathological pregnancies were significantly higher than DS pregnancies (Figure 6b); however, free hCGbeta concentrations, which positively correlate with total serum hCG (r, 0.54, data not shown), were significantly higher in DS compared to control and pathological pregnancies.

## Discussion

Here we describe the development of and use of two ELISA systems for measuring circulating, soluble hCG receptor (sLHCGR) and hCG-receptor complex (hCG-sLHCGR) in sera from early human pregnancies. The specificity, sensitivity and accuracy of these assays were established using recombinant protein calibrators and poly-/monoclonal antibodies. Both ELISAs were equally effective in measuring sLHCGR/hCG-sLHCGR in frozen serum or plasma samples stored at −20°C for up to 10 years. The sensitivities of the sLHCGR and hCGbeta-sLHCGR assays described here were insufficient to detect analytes in about 4.6% of the early pregnancy samples. The current sensitivity (0.9-1.2 pmol/mL) of these assays could be improved by replacing the HRP-tag on the detection antibody with chemi-luminescent or fluorescent-labels.

### sLHCGR - a unique biomarker with low and high cut-off values

Quantitative analysis of sLHCGR and hCG-sLHCGR complex in early human pregnancy serum samples showed a wide spectrum of circulating receptor concentrations: 0 to >3,500 pmol/mL. By correlation with pregnancy outcome, it was observed that pregnancies with a poor outcome had either very low (≤5 pmol/mL) or significantly high (≥170 pmol/mL) concentrations of hCGbeta-sLHCGR, in combination with low PAPP-A (<0.8 MoM). Unlike other pregnancy biomarkers, sLHCGR appears to be unique due to two cut-off (low and high) values. While this may reflect the placental and extra-placental origin of the receptor, it may also be indicative of a physiological function for sLHCGR that is defined by concentration range during normal pregnancy. The almost ubiquitous expression of LHCGR in the reproductive organs and certain extragonadal tissues [2,22] is consistent with the former explanation. Notably, pregnancies that suffered fetal demise/stillbirth following first trimester screen only had undetectable and low serum sLHCGR. These observations could be clinically applied by using serum sLHCGR/hCG-sLHCGR concentrations to predict abnormal outcome at very early human pregnancy. For example, low PAPP-A (≤0.5 MoM) in combination with high hCGbeta ≥ 2.0 MoM) are positive in the primary screening for Down’s syndrome (21). However, >20% of the Down’s serum samples (9 of 43) with low PAPP-A (≤0.5 MoM) were only detectable in combination with hCG-sLHCGR. Therefore, the addition of serum hCG-sLHCGR measurement to the current Down’s screening protocol, that uses a combination of PAPP-A and free hCGbeta measurement, would increase the biochemical detection rate of Down’s syndrome. With respect to preeclampsia, premature birth and fetal demise, measurement of hCGbeta has very little or no diagnostic application [23]. We have demonstrated that hCG-sLHCGR plus PAPP-A measurement can identify pregnancies with an adverse outcome which are chromosomally normal. However, larger population-based studies, involving both low and high-risk groups, are required to establish the cut-off values for serum hCG-sLHCGR/sLHCGR. Furthermore, the diagnostic potential of sLHCGR systems in combination with PAPP-A and other early pregnancy markers needs to be established.

### Possible mechanistic basis for the secretion of low and high sLHCGR

With respect to serum hCG-sLHCGR concentrations, undetectable or low levels could indicate placental insufficiency, reduced angiogenesis and perfusion at very early pregnancy. The majority of pregnancies that are Down’s or preeclampsia, or those leading to miscarriage and fetal demise, belong to this category. Low or undetectable serum hCG-sLHCGR could be linked to pregnancy pathology by two distinct alternative mechanisms. In the first mechanism, low levels of sLHCGR may reflect placental deficiency of the cell-surface bound receptor (low LHCGR expression) and consequent disruption of physiological hCG signaling. This view is supported by studies showing a dramatic reduction of full-length LHCGR expression in Down’s syndrome chorionic villi compared to chromosomally normal pregnancies [18,24]. It should be noted, however, that the present study failed to detect any sLHCGR/hCG-sLHCGR in about 4.6% of the pregnancies and yet, many of these pregnancies have both normal PAPP-A and free hCGbeta and resulted in live births with no obvious adverse outcomes for either mother or baby. One possible explanation could be that hCG variants in different individuals modulate the receptor dynamics with respect to its release from the tissues as well as its clearance from the circulation. All sera analysed in this study were from 10–13 wk gestation. It is possible that sLHCGR (and hCG-sLHCGR) may have more meaningful diagnostic potential earlier (<10 wks) in the first trimester.

In the second mechanism, elevated circulating hCG receptor at early pregnancy might represent placental pathology (reduced placental perfusion and oxidative stress) in response to the pro-inflammatory mediators leading to pathological secretion of sLHCGR. A significant number of Down’s pregnancies, preterm delivery and preeclampsia pregnancies (the present study and unpublished data) belong to this category. Very high levels of serum sLHCGR might also be linked to reduced clearance of hCG when serum concentrations of this hormone are high. Notably elevated levels of serum hCG in the second trimester are associated with preeclampsia [25-29]. The ligand induced secretion of M_r_ 80K-90K hCG-sLHCGR complexes into the culture media (8) further suggests hormonal regulation of LHCGR secretion. The impact of high serum sLHCGR in pregnancy could lead to reduced hCG bioactivity [29], aberrant systemic vasculo-endothelial and immune activation [30-32]. In normal pregnancies sLHCGR may act as a reservoir for easily available hCG. It is interesting to note that there is a 15-fold difference between the lowest and highest levels of total hCG that are considered normal in first trimester pregnancy (20-300K IU/L). The present study shows an interesting parallel in that normal pregnancies have a wide range of sLHCGR levels, with a minimum of a 17-fold difference between low and high (between 10 pmol/mL and 170 pmol/mL).

### Could high sLHCGR affect thyroid function in pregnancy?

There could also be a role for serum sLHCGR in modulating thyroid hormone. High concentrations of hCG (>50,000 IU/L) at early pregnancy have a thyrotropic effect in about 18% of pregnancies [4,33,34]. By virtue of its similarity to the catalytic subunit of thyroid stimulating hormone (TSH), hCG transiently suppresses the TSH level and functionally replaces the TSH by directly activating the TSH receptor in thyroid epithelium [35]. However, TSH could potentially bind sLHCGR (via a non-cognate interaction, see ref 3) provided that the free sLHCGR (unbound to hCG) concentration is high (spill-over effect). About 5% of all normal outcome pregnancies tested in the present study have very high sLHCGR. Therefore, a potential sLHCGR concentration-dependent binding of TSH in these pregnancies could have two-fold effects: Inhibition of the thyrotropic effect of TSH and simultaneous increase in half life of TSH (hypothyroidism). Further studies are needed to examine whether subclinical hypothyroidism, which is strongly associated with preeclampsia [36-38], is linked to high serum sLHCGR levels and relatively low total hCG levels, leading to unsaturated sLHCGR at early pregnancy. Moreover, it has been well established that up to 12 wks of gestation, the maternal thyroxin is the major source for activating the fetal CNS receptors. Therefore, extremely high sLHCGR and low hCG in a very small set of pregnancies (<2%, Figure 6b) could be linked to impaired fetal brain development [39] at the first trimester of pregnancy.

### Possible physiological role for sLHCGR

The two extreme ends of the spectrum of serum soluble hCG receptor concentrations representing adverse pregnancy outcome (Figures 5 and 6), suggests intermediate levels of sLHCGR might be important in regulating hCG signaling in normal pregnancy. Microvesicle-associated LHCGR, released from the placenta [12] could facilitate hCG signaling at early pregnancy. Microvesicles released from the tissues, upon fusion with distant target tissues could transiently confer new properties by reprogramming the recipient cells such as maternal vasculo-endothelial (VE), endometrial epithelial cells (EEC) and immune cells at the fetal-maternal interface [31]. Exosomes are constitutively secreted by placental syncytotrophoblasts throughout the pregnancy [40]. Placental exosome-mediated transduction of the ligands for the receptors on the surface of maternal cytotoxic T-cells (Fas-FasL) and NK cell (NKG2D receptor-ligand) is pivotal to eliciting immune tolerance at early gestation [41,42]. Moreover, trophoblasts stimulate endometrial angiogenesis by two alternative mechanisms: Direct activation of endothelial cells by hCG and indirect paracrine regulation of EEC by hCG, stimulating the secretion of VEGF [30-32]. Microvesicles bearing sLHCGR, following transfer of the receptor to uterine smooth muscle, EC and EEC, could enhance existing cell-surface receptor hCG signaling pathways critical for uterine capillary formation and increased arterial blood flow. Therefore, reduced secretion of placental sLHCGR could interfere with utero-placental cross-talk at very early pregnancy. The second physiological role of sLHCGR could be increasing the half-life of the hormone hCG by stabilizing the hormone-receptor complex for controlled hCG signaling in the target tissues.

## Conclusions

Two novel immunoassays for measuring serum/plasma sLHCGR and hCG-sLHCGR complex in human pregnancy have been developed and validated. Clinical evaluation, using serum samples from first trimester pregnancies, revealed that quantitative analysis of soluble hCG receptor/ hCG-sLHCGR, together with existing analytes (free hCGbeta and PAPP-A) could significantly improve early detection of pregnancy pathology.

## Competing interests

The use of sLHCGR-based immunodiagnostic tests has been patented.

## Authors’ contributions

AEC developed and validated the ELISA system, performed most of the ELISAs, analyzed the data, contributed to the interpretation of results, creation of manuscript figures and first draft of the manuscript. CG collected whole blood, liased with consenting patients and provided clinical diagnoses based on ultrasound, biochemical (PAPP-A and hCGbeta) and molecular analyses. WEM performed statistical analysis on the data, contributed to the creation of manuscript figures and to the interpretation of the results. IM and SAN prepared and stored sera from whole blood, performed biochemical analyses (PAPP-A and hCGbeta) and collected clinical data. AS and KHN collected, prepared and stored sera, performed clinical diagnoses and provided clinical data. SB conceived and initiated the study, performed some ELISAs, including those for validation, did all western blotting, designed the standards and monoclonal antibodies used in the ELISAs, presided over the statistical analyses, interpretation of results, creation of manuscript figures and the first draft of the manuscript. All authors read and approved the final manuscript.
